# Long-term effect of latanoprost/timolol fixed combination in patients with glaucoma or ocular hypertension: A prospective, observational, noninterventional study

**DOI:** 10.1186/1471-2415-10-21

**Published:** 2010-09-08

**Authors:** Oliver Schwenn, Barbara Heckmann, Claudia Guzy, Paul J Miller

**Affiliations:** 1 Bürgerhospital Frankfurt am Main, Augenklinik, Frankfurt am Main, Germany; 2 Private practice, Bruchsal, Germany; 3 Pfizer Pharma GmbH, Berlin, Germany; 4 Pfizer Limited, UK Specialty Care, Sandwich, Kent, UK

## Abstract

**Background:**

Prospective, observational studies that enroll large numbers of patients with few exclusion criteria may better reflect actual ongoing clinical experience than randomized clinical trials. Our purpose was to obtain efficacy and safety information from a cohort of subjects exposed to latanoprost/timolol fixed combination (FC) for ≥18 months using a prospective, observational design.

**Methods:**

In all, 577 office-based ophthalmologists in Germany switched 2339 patients with glaucoma or ocular hypertension to latanoprost/timolol FC for medical reasons. Follow-up visits were scheduled for every 6 months over 24 months; physicians followed usual care routines. Intraocular pressure (IOP), visual field status, optic nerve head findings, and adverse events were recorded. Efficacy parameters were evaluated for the per protocol (PP) population; the safety population included subjects receiving ≥1 drop of FC. Physicians rated efficacy, tolerability, and subject compliance at month 24.

**Results:**

Of the 2339 subjects switched to latanoprost/timolol FC (safety population), the primary reasons for switching were inadequate IOP reduction (78.2%) and desire to simplify treatment with once-daily dosing (29.4%; multiple reasons possible). In all, 1317 (56.3%) subjects completed the study, and 1028 (44.0%) were included in the PP population. Most discontinuations were due to loss to follow-up. Change in mean IOP from baseline to month 6 was -4.0 ± 4.31 mmHg, a reduction that was maintained throughout (P < 0.05 for change at all time points). By investigator assessments, optic disc parameters and visual field were stable over 24 months, and there was no relationship between IOP reduction over 24 months and development of a visual field defect. More than 90% of physicians rated latanoprost/timolol FC as "very good" or "good" for efficacy (PP population), tolerability, and compliance. The FC was safe and well tolerated. No change in iris color was reported by most subjects (83.1%) at month 24.

**Conclusions:**

Over 24 months, latanoprost/timolol FC effectively lowers IOP levels and is well tolerated in patients with glaucoma or ocular hypertension who change from their previous ocular hypotensive therapy for medical reasons. Investigator assessments found optic disc parameters and visual field to be stable throughout 24 months of follow-up.

## Background

In patients with glaucoma or ocular hypertension who do not reach their target intraocular pressure (IOP) level with ocular hypotensive monotherapy, the European Glaucoma Society [1] recommends adding a second medication when the original agent showed some effectiveness. In fact, many patients with these conditions must use more than one ocular hypotensive therapy to reduce IOPs to levels that may be expected to slow or stop disease progression [2]. In these individuals, a fixed-combination (FC) formulation may be preferred to multidrug regimens in order to maximize patient compliance and quality of life [1].

In Europe, the FC of the prostaglandin analogue latanoprost and the beta-blocker timolol is approved for the treatment of open-angle glaucoma or ocular hypertension in patients insufficiently controlled on monotherapy. Latanoprost, the first prostaglandin F_2a _analogue to be commercially available in Europe and the United States, acts primarily by increasing outflow [3,4] while the beta-adrenergic receptor antagonist timolol lowers IOP by reducing aqueous humor production [5,6]. The combination of the two agents has been shown to have an additive IOP-lowering effect [7-10], and several prospective, randomized clinical trials have demonstrated that latanoprost/timolol FC is effective and well tolerated [11-16].

Although such prospective, randomized trials are the gold standard for evaluating the safety and efficacy of new medical treatments, their strict designs may not reflect community practice patterns thereby limiting the generalizability of findings. Prospective, observational studies that include large numbers of patients with few exclusion criteria may better reflect actual ongoing clinical experience. The present prospective, noninterventional, observational study was designed to obtain efficacy and tolerability information about a cohort of subjects exposed to the latanoprost/timolol FC for at least 18 months.

## Methods

The study was conducted in general ophthalmology practices in Germany between August 2005 and December 2008. The study met all standards for ethical approval in Germany. It was planned and conducted and data were analyzed in accordance with guidelines issued by the Bundesinstitut für Arzneimittel und Medizinprodukte (Federal Institute for Drugs and Medical Devices). German law does not require patient informed consent in observational studies in which treatment is medically indicated by the physician regardless of study participation and in which treatment use is restricted to approved indications.

### Procedures and measurements

In all, 577 office-based ophthalmologists treated and provided information for 2339 subjects with glaucoma or ocular hypertension who were being switched for medical reasons to once-daily latanoprost/timolol FC from another ocular hypotensive medication (monotherapy, FC, or unfixed combination). Participating ophthalmologists followed their usual care practices. At the baseline visit, the reason(s) for switching the subject to latanoprost/timolol FC was noted, and demographic data, medical and ocular histories, visual field status (Aulhorn stage and mean defect), and optic nerve head findings were recorded. Prior to pupil dilation, best-corrected visual acuity was determined, and IOP level was measured once by pulse air tonometry or calibrated Goldman applanation tonometry.

Study-related follow-up visits were scheduled to occur at approximately 6-month intervals for 24 months. At each visit, IOP was measured, optic disc excavation and visual field (Aulhorn stage) were assessed, and glaucoma damage/progression was evaluated by investigators. Any decision to withdraw FC therapy before 24 months was made at the discretion of the treating physician.

All observed or volunteered adverse events and serious adverse events were recorded throughout the study. Serious adverse events were those that were life-threatening, resulted in death, required or prolonged hospitalization, or resulted in disability or congenital anomaly. Suspected causal relationships to latanoprost/timolol FC were recorded by treating physicians. Version 12.0 of the Medical Dictionary for Regulatory Activities (MedDRA) was used to code diagnoses, previous/concomitant diseases, and adverse events.

Physicians assessed the overall efficacy and the overall tolerability of latanoprost/timolol FC at month 24 as "very good", "good", "moderate", or "insufficient". Subject compliance with the FC was evaluated by physicians using the same four categories. At month 24, subjects evaluated change in iris color from baseline as "none", "slightly", "distinctly", or "very distinctly" and were asked whether they wished to remain on the FC.

### Endpoints and analyses

Statistical analyses were descriptive and exploratory. Percentages for categorical variables as well as means, standard deviations (SDs), and, where appropriate, two-sided 95% confidence intervals (CIs) for continuous variables were calculated based on nonmissing observations. Associations between pairs of variables were assessed using Pearson correlation for continuous variables, Spearman rank correlation where one or both variables were ordinal, or tetrachoric correlation for two binary variables.

If both eyes were treated with the FC, the IOP value for the right eye was used; otherwise, the value for the treated eye was used. If the physician did not indicate which eye was treated, it was assumed that both eyes were prescribed FC therapy. Mean changes in IOP levels at months 6, 12, 18, and 24, and at the last visit were assessed. In the analysis of changes in IOP, the last visit was defined as the last postbaseline visit at which an IOP level was recorded. In addition, mean change in corrected IOP from baseline to last visit was assessed using the formula developed by Kohlhaas et al. [17] (corrected IOP = raw IOP + [-0.0423 × central corneal thickness in μm + 23.28]), and for the subset of subjects in whom IOP was measured using applanation tonometry and for subjects stratified by diagnosis and by baseline ocular hypotensive therapy.

Mean changes from baseline in horizontal and vertical cup/disc ratios were evaluated across visits. Aulhorn stage and mean defect at each visit and change in stage from baseline were summarized. A stepwise analysis of variance (ANOVA) of mean change in visual field defect (last visit at which the parameter was recorded - baseline) included the following potential explanatory variables: age, gender, baseline mean defect, change in IOP (last visit at which the parameter was recorded - baseline), number of postbaseline optic disc hemorrhages, treatment duration, primary diagnosis, history of hypertension, history of hypotension, and history of diabetes. The significance level for variable entry was set at 0.05 and for retention at 0.10; no interaction terms were fitted.

A 6-item composite variable reflecting progression of glaucomatous damage from baseline to last visit was defined as any of the following: (a) increase in horizontal or vertical cup/disc ratio by ≥0.2; (b) occurrence of ≥1 postbaseline optic disc hemorrhage; (c) decrease in rim area, rim volume, and/or mean retinal nerve fiber layer thickness by 0.2 mm^2^, 0.1 mm^3 ^and 0.1 mm, respectively, as measured by Heidelberg Retina Tomograph; (d) progressive visual field deterioration noted by the physician at ≥1 postbaseline visit; (e) increase in Aulhorn stage by ≥1 stage; or (f) decrease in mean defect by ≥2.5 dB. A stepwise logistic regression analysis of the binary variable presence/absence of progression included the following potential predictors: age, gender, baseline IOP, change in IOP (last visit - baseline), primary diagnosis, history of hypertension, history of hypotension, and history of diabetes. The significance level for variable entry was set at 0.05 and for retention at 0.10; no interaction terms were fitted. In addition, progression of optic disc excavation (present if either criteria [a] or [c], above, was met) and progression of visual field (present if criteria [d] and if criteria [e] and/or [f] were met) were evaluated.

Efficacy parameters were analyzed for the per protocol (PP) population which included subjects treated with latanoprost/timolol FC for ≥18 months who had a baseline and ≥1 postbaseline IOP measurement (≥18 months apart), did not have a refractive error ≤ -8 diopters or ≥ + 8 diopters at baseline, and did not administer ocular hypotensive medication in addition to latanoprost/timolol FC medication during the study period. This definition of the PP population was appropriate given that the primary objective of this noninterventional study was to obtain information about a cohort of subjects exposed to the latanoprost/timolol FC for at least 18 months. In addition, key efficacy analyses were reproduced using the full analysis set (FAS) population, which included all subjects with ≥1 postbaseline IOP measurement, in order to evaluate the robustness of the PP analyses. The safety population included all subjects who received ≥1 drop of study medication.

## Results

Of the 2339 subjects switched to latanoprost/timolol FC (safety population), 1317 (56.3%) completed the study. Subject disposition is summarized in Table 1. A total of 1022 subjects (43.7%) discontinued from the study; the vast majority of discontinuations (894) were unrelated to study drug, and nearly all of those (851) were attributable to loss to follow-up. In all, 1028 subjects met criteria for inclusion in the PP population, and 1934 were eligible for the FAS population.

**Table 1:** Subject disposition

	No. (%) subjects
Received latanoprost/timolol FC	2339
Completed 24 months of follow-up	1317 (56.3)
Discontinued prior to 24 months	1022 (43.7)
Per protocol population	
Included	1028 (44.0)
Excluded	1311 (66.0)
Reason(s) for exclusion:*	
Not treated for ≥18 months	512
No baseline and ≥1 postbaseline measure for IOP ≥18 months apart	487
Additional ocular hypotensive medication during study	402
Ametropy at baseline	42
Full analysis set	
Included	1934 (82.7)
Excluded (no postbaseline IOP measurement)	405 (17.3)
Safety population	2339 (100.0)

FC = fixed combination; IOP = intraocular pressure.

*More than one reason was possible. If a subject was excluded from the full analysis set, a separate reason for exclusion from the per protocol population is not provided in this table.

In the total study population, the average age was 65.5 years, and 1047/2339 (44.8%) subjects were male (Table 2). The most common primary diagnoses were open-angle glaucoma and ocular hypertension. The ocular hypotensive therapies most often reported at the time of the switch to the FC were latanoprost (n = 343), timolol (n = 173), and timolol maleate (n = 115). (Investigators could report the same drug as "timolol" or "timolol maleate" reflecting different preferences in drug terms.) The most commonly reported reasons for switching to the FC were inadequate IOP reduction on prior therapy (78.2%) and desire to simplify treatment with once-daily dosing (29.4%; Table 2). The median duration of latanoprost/timolol FC treatment was 708 days with 1491/2339 (63.7%) subjects treated with the FC for at least 18 months.

**Table 2:** Baseline characteristics, N = 2339

Age (years)*	
Mean ± SD	65.5 ± 11.7
Range	10, 96
Male gender, n (%)*	1047 (44.8)
Primary diagnosis^†^	
Open-angle glaucoma	1910
Ocular hypertension	177
Pseudoexfoliation glaucoma	114
Normal-tension glaucoma	111
Glaucoma NOS	41
Angle-closure glaucoma	17
Pigmentary glaucoma	12
Ocular hypotensive therapy reported by ≥50 subjects	
Bimatoprost	50
Brinzolamide	92
Dorzolamide/timolol FC	92
Latanoprost	343
Timolol	173
Timolol maleate	115
Travoprost	93
Reason(s) for switching to latanoprost/timolol FC,^‡ ^n (%)	
Inadequate IOP reduction on prior therapy	1830 (78.2)
Desire to simplify treatment with once-daily dosing	687 (29.4)
Side effects/hypersensitivity reactions with prior therapy	219 (9.4)
Prior therapy contraindicated due to subject's signs/symptoms	103 (4.4)
Other	120 (5.1)

FC = fixed combination; IOP = intraocular pressure; NOS = not otherwise specified; SD = standard deviation.

*Age was missing for 57 subjects; gender was missing for 48 subjects.

^†^Counts are based on diagnoses for both eyes. If a subject had a different diagnosis for each eye, these were counted twice, and if a subject had more than one diagnosis for the same eye, each was counted.

^‡^More than one reason could have been recorded by physicians. Percentages are based on the number of patients (N = 2339).

**Figure 1:**
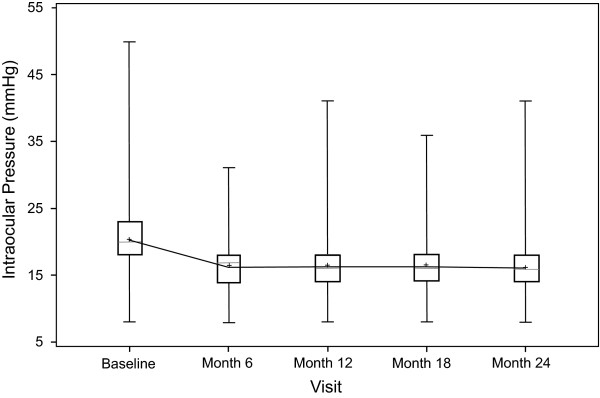
**Box-plot of intraocular pressure by visit, PP population**. PP = per protocol. Bars represent minimum and maximum values. Boxes represent interquartile range and median. Line presents the mean profile over time.

In the PP population, the mean baseline IOP was 20.3 ± 4.20 mmHg (Table 3). A mean change from baseline of -4.0 ± 4.31 mmHg was noted at month 6; this decrease was maintained and reductions were statistically significant throughout the follow-up period (Table 3; Figure 1; P < 0.05 for each change from baseline). Similar reductions from baseline to last visit were noted when IOP values were corrected using the formula proposed by Kohlhaas et al. [17]; in the FAS population; and among the more than 600 subjects in the PP population whose IOP levels were evaluated by applanation tonometry (Table 4). With the PP population stratified by primary diagnosis, mean ± SD changes from baseline to last visit in IOP levels were -4.1 ± 4.34 mmHg in the open-angle glaucoma group (n = 859), -4.6 ± 4.04 mmHg among those with ocular hypertension (n = 83), -5.1 ± 6.78 mmHg in pseudoexfoliation glaucoma subjects (n = 48), and -3.2 ± 3.75 mmHg in subjects with normal-tension glaucoma (n = 50). Stratified by previous ocular hypotensive medication, mean change in IOP from baseline to last visit was ≥-4.0 mmHg for those previously treated with a monotherapy or with a single FC therapy; the mean ± SD IOP reduction in subjects switched to latanoprost/timolol FC from multiple therapies was -2.5 ± 4.48 mmHg (n = 119) and was -4.5 ± 4.77 mmHg in subjects for whom the prior ocular hypotensive therapy was not recorded (n = 586).

**Table 3:** Mean IOP* and mean change in IOP from baseline^† ^by visit (mmHg), PP population

Visit	n	Mean ± SD (95% CI)	Change from baseline, mean ± SD **(95% CI**^‡^)
Baseline	1028	20.3 ± 4.20	n.a.
		(20.1, 20.6)	
Month 6	1012	16.4 ± 3.04	-4.0 ± 4.31
		(16.2, 16.6)	(-4.3, -3.8)
Month 12	1017	16.4 ± 3.04	-3.9 ± 4.53
		(16.2, 16.6)	(-4.2, -3.7)
Month 18	1010	16.4 ± 2.99	-4.0 ± 4.43
		(16.2, 16.5)	(-4.3, -3.7)
Month 24	980	16.2 ± 3.17	-4.2 ± 4.69
		(16.0, 16.4)	(-4.5, -3.9)
Last visit^§^	1028	16.2 ± 3.16	-4.1 ± 4.66
		(16.0, 16.4)	(-4.4, -3.8)

CI = confidence interval; IOP = intraocular pressure; n.a. = not applicable; PP = per protocol;

SD = standard deviation.

*Raw IOP values.

^†^Month × - baseline.

^‡^P < 0.05 for change from baseline at each visit.

^§^Last visit = last postbaseline visit at which an IOP level was recorded.

**Table 4:** Mean IOP and mean change in IOP from baseline to last visit* (mmHg), alternate populations

Visit	n	Mean ± SD (95% CI)	Change from baseline, mean ± SD **(95% CI**^†^)
**PP population: corrected IOP**[17]			
Baseline	546	20.5 ± 3.94	n.a.
		(20.2, 20.9)	
Last visit	546	16.3 ± 3.30	-4.2 ± 4.21
		(16.0, 16.6)	(-4.6, -3.9)
**Full analysis set: raw IOP**			
Baseline	1913	20.4 ± 4.25	n.a.
		(20.2, 20.6)	
Last visit	1934	16.4 ± 3.42	-3.9 ± 4.65^‡^
		(16.3, 16.6)	(-4.2, -3.7)
**PP population with applanation tonometry data: raw IOP**			
Baseline	732	20.3 ± 4.31	n.a.
		(20.0, 20.6)	
Last visit	687	16.2 ± 3.02	-4.0 ± 4.59^§^
		(16.0, 16.4)	(-4.3, -3.6)

CI = confidence interval; IOP = intraocular pressure; n.a. = not applicable; PP = per protocol;

SD = standard deviation.

*Last visit = last postbaseline visit at which an IOP level was recorded.

^†^P < 0.05 for change from baseline to last visit for each parameter.

^‡^N = 1913.

^§^N = 631.

Changes from baseline in horizontal and vertical cup/disc ratios showed a tendency toward stability and were not considered to be clinically significant (Table 5). Among subjects in the PP population for whom information concerning whether or not they had an optic disc hemorrhage at any postbaseline visit was available, the percentage with a hemorrhage was lower at each follow-up visit than at baseline (20/916 [2.2%] at baseline vs ≤1.5% at any postbaseline visit). In all, 29/983 (3.0%) reported ≥1 postbaseline optic disc hemorrhage, and 4/983 (0.4%) had repeated occurrences at >1 postbaseline visit.

**Table 5:** Mean change from baseline* in horizontal and vertical cup/disc ratios by visit, PP population

Visit	n	Mean ± SD (95% CI)
**Horizontal cup/disc ratio**		
Month 6	645	0.0003 ± 0.13117
		(-0.0099, 0.0104)
Month 12	660	0.0039 ± 0.13286
		(-0.0063, 0.0140)
Month 18	666	0.0092 ± 0.13860
		(-0.0014, 0.0197)
Month 24	666	0.0041 ± 0.14078
		(-0.0067, 0.0148)
Last visit	783	0.0079 ± 0.14645
		(-0.0024, 0.0182)
**Vertical cup/disc ratio**		
Month 6	600	0.0040 ± 0.12772
		(-0.0062, 0.0142)
Month 12	604	0.0075 ± 0.13726
		(-0.0035, 0.0184)
Month 18	613	0.0152 ± 0.14613
		(0.0036, 0.0268)
Month 24	615	0.0088 ± 0.13534
		(-0.0019, 0.0195)
Last visit	729	0.0127 ± 0.15155
		(0.0017, 0.0237)

CI = confidence interval; PP = per protocol; SD = standard deviation.

*Month × - baseline. Last visit = last postbaseline visit at which the parameter was recorded

Information concerning mean visual field defect measurement method was available for 744 subjects, and the most commonly used measurement method was the Humphrey Visual Field Analyzer (n = 337). At month 24, 72 of the 122 subjects (59.0%) with valid data for the Aulhorn stage based on the Humphrey Visual Field Analyzer at both baseline and month 24 had no change in stage; 15.6%, 2.5% and 0.8% of subjects increased by 1, 2 and 3 stages, respectively, whereas 15.6%, 4.9%, 0.8% and 0.8% of subjects decreased by 1, 2, 3, and 4 stages, respectively.

In the PP population, there were no statistically significant changes in mean defect from baseline to months 6, 12, 18, and 24 or to the last visit. In the multivariate analysis, change in mean defect from baseline to the last visit was related only to baseline mean defect. This analysis only involved subjects with nonmissing data for the response variable and all potential explanatory variables (n = 355). The final model was reduced to a simple linear regression with intercept -0.718 and slope -0.283 (95% CI: -0.354, -0.211; P < 0.0001). Hence, subjects with a lower baseline mean defect experienced a higher change in mean defect from baseline to last visit. In addition, the correlation between changes from baseline to last visit in mean defect and in IOP was estimated at 0.0276 (95% CI: -0.0603, 0.1156; P = 0.5956; n = 371), providing no evidence of a relationship between IOP reduction and reduction in mean defect.

Fewer than 18% of evaluable subjects demonstrated a negative change in any individual progression measure by last visit or month 24 (Table 6). By the last visit, progression of optic disc excavation (increase in horizontal or vertical cup/disc ratio by ≥0.2 or decrease in rim area, rim volume, and/or mean retinal nerve fiber layer thickness by 0.2 mm^2^, 0.1 mm^3 ^and 0.1 mm, respectively, as measured by Heidelberg Retina Tomograph) was noted in 117/816 (14.3%) subjects. Visual field progression (progressive visual field deterioration noted by the physician at ≥1 postbaseline visit and an increase in Aulhorn stage by ≥1 stage and/or decrease in mean defect by ≥2.5 dB) occurred in 46/900 (5.1%) of subjects by the last visit. Based on the six-item composite variable, overall progression of glaucoma by the last visit was noted in 305 (30.2%) of the 1010 subjects in the PP population who provided a response to ≥1 of the six individual progression measures. Logistic regression demonstrated that only age was a significant predictor of composite overall progression by the last visit (odds ratio = 0.984; 95% CI: 0.972, 0.996; P = 0.0102; n = 952). From this final model, there is evidence that the probability of experiencing overall progression of glaucoma damage increases with age.

**Table 6:** Changes in progression measures by last visit* and month 24, n/N (%), PP population

Progression measure	Last visit	Month 24
Increase in horizontal or vertical cup/disc ratio by ≥0.2 (visit - baseline)^†^	98/797 (12.3)	78/676 (11.5)
≥1 postbaseline optic disc hemorrhage^‡^	29/983 (3.0)	23/785 (2.9)
Decrease in ≥1 rim area, rim volume, and/or mean RNFL thickness by HRT (visit - baseline)^†^	28/222 (12.6)	13/151 (8.6)
Visual field deterioration rated as progression by physician at ≥1 postbaseline visit^‡^	137/884 (15.5)	104/630 (16.5)
Increase in Aulhorn stage^§ ^by ≥1 stage (visit - baseline)^†^	64/370 (17.3)	43/258 (16.7)
Decrease in mean defect by ≥2.5 dB^†^	59/371 (15.9)	45/285 (15.8)

HRT = Heidelberg Retina Tomograph; PP = per protocol; RNFL = retinal nerve fiber layer.

*Last visit = last postbaseline visit at which the parameter was recorded.

^†^To be included in the percentage, a subject must have had assessments at both the baseline and ≥1 postbaseline visit for the relevant measure.

^‡^To be included in the percentage, a subject must have had ≥1 postbaseline assessment for the relevant measure.

^§^Reflects all methods of determining Aulhorn stage.

Latanoprost/timolol FC was safe and well tolerated. Sixteen deaths were reported, none of which was considered by investigators to be related to study treatment. In all, 148 subjects treated with latanoprost/timolol FC experienced 185 adverse events, and 88 treatment-related adverse events were reported in 72 subjects (Table 7). Three (0.1%) subjects experienced serious adverse events deemed by investigators to be related to study treatment. Fifty-two (2.2%) subjects permanently discontinued treatment with the FC due to a treatment-related adverse event. Ocular adverse events were the most commonly reported adverse events. Treatment-related ocular adverse events were noted in 39 (1.7%) subjects, and 28 (1.2%) permanently discontinued FC therapy due to such an event. Two (0.1%) subjects experienced a serious treatment-related ocular adverse event. Most adverse events resulting in discontinuation were mild or moderate in severity and resolved by the end of the study.

**Table 7:** Adverse events, N = 2339

	All causalities n (%)	Treatment related n (%)
**All adverse events**		
Number of events	185	88
Subjects with:		
≥1 adverse event	148 (6.3)	72 (3.1)
≥1 serious adverse event	54 (2.3)	3 (0.1)
≥1 severe adverse event	70 (3.0)	15 (0.6)
Discontinued FC due to adverse event	69 (2.9)	52 (2.2)
Dose reduced/temporarily discontinued FC due to adverse event	22 (0.9)	8 (0.3)
**All Ocular Adverse Events**		
Number of events	99	44
Subjects with:		
≥1 adverse event	84 (3.6)	39 (1.7)
≥1 serious adverse event	29 (1.2)	2 (0.1)
≥1 severe adverse event	35 (1.5)	5 (0.2)
Discontinued FC due to adverse event	41 (1.8)	28 (1.2)
Dose reduced/temporarily discontinued FC due to adverse event	19 (0.8)	6 (0.3)

FC = fixed combination.

At month 24, the majority of responding physicians rated the overall efficacy of latanoprost/timolol FC as "very good" or "good" (PP population: 922/997 [92.5%]; FAS population: 1312/1504 [87.2%]). Among subjects for whom physicians provided evaluations at month 24, the overall tolerability of the FC was assessed as either "very good" or "good" in 1503/1584 (94.9%) subjects and compliance with the FC as "very good" or "good" in 1439/1580 (91.1%) subjects. More than three-quarters (1123/1351 [83.1%) of responding subjects reported no change in iris color at month 24, and nearly 90% (n = 1343/1520 [88.4%]) indicated a desire to continue FC treatment after the completion of study. At month 24, change in visual acuity from baseline was not statistically or clinically significant.

## Discussion

Results of this long-term observational study of latanoprost/timolol FC demonstrate that the combination effectively reduces IOP levels and is well tolerated in patients with glaucoma or ocular hypertension who change from their previous ocular hypotensive therapy for medical reasons. The significant IOP-lowering effect of the FC was seen early, at the month 6 visit, and was sustained throughout the 24-month follow-up period. Moreover, no significant changes in optic disc and visual field defect were noted by investigators, and structural and functional parameters remained stable over 24 months. Investigator assessments revealed no significant association between IOP reduction over two years and change in visual field.

Previous research has demonstrated that progression of glaucoma or ocular hypertension can be delayed or halted by lowering IOP levels through the use of ocular hypotensive agents [2,18-21]. Herein, the mean IOP reduction of approximately 4 mmHg sustained over 24 months was somewhat greater than reductions reported in previous short-term observational studies of patients switched to the FC [22,23]. For example, a prospective, multicenter study [22] of patients switched to latanoprost/timolol FC and followed for at least two months found mean IOP reductions from baseline of 2.9 mmHg in those with primary open-angle glaucoma or exfoliation glaucoma and of 3.1 mmHg among patients with ocular hypertension (P < 0.001 for all). A multicenter, observational study [23] of patients with glaucoma or ocular hypertension who were switched to the FC and followed for six months reported mean IOP reductions from baseline of 3.3 mmHg, 4.1 mmHg, and 3.4 mmHg among patients with open-angle glaucoma, exfoliation glaucoma, and ocular hypertension, respectively (P < 0.001 for all).

As has been shown previously [11-16], the FC was well tolerated. In all, 99/185 (53.5%) adverse events were ocular in nature, half of treatment-related adverse events related to the eye, and two of the three reported treatment-related serious adverse events were ocular. Fewer than 3% of subjects discontinued the FC due to an ocular adverse event. The tolerability of an ocular hypotensive agent is important given the negative impact of ocular adverse events on patient continuation with therapy [24,25].

At month 24, physician evaluations of latanoprost/timolol FC were overwhelmingly positive with regard to efficacy, tolerability, and patient compliance. In addition, nearly 90% of patients expressed a desire to remain on the FC after the end of the study. These positive evaluations are tempered, however, by the fact that they were made for and by patients who stayed on therapy for the full follow-up period; it is not known how many of those for whom efficacy, tolerability, or compliance were issues discontinued FC therapy prior to that time point. Conversely, nearly 90% of discontinuations in the present study were unrelated to latanoprost/timolol FC, with the vast majority attributable to loss to follow-up. Moreover, a prior study [23] of patients switched to latanoprost/timolol FC found that 97% of patients (1008/1042) remained on treatment after the 6-month study period.

Benefits of prescribing a FC agent for patients with glaucoma or ocular hypertension may include improved adherence, persistence, convenience, and reduced exposure to preservatives. Improved adherence and persistence, in particular, are critical since the use of an effective ocular hypotensive agent over the long term may be expected to increase the likelihood of delaying or stopping glaucomatous damage. Poorer compliance has been demonstrated in those treated with more complex medication regimens [26-30]. Comparative studies of medication compliance in patients prescribed alternative FC therapies are needed.

This study has both strengths and limitations. The observational design may have better reflected actual clinical practice than controlled clinical trials, but the absence of a control group limits our ability to draw conclusions, and the PP population included fewer than half the number of subjects treated, primarily due to loss to follow-up. Given the observational design, it was not possible to standardized the timing and method of measuring IOP levels and visual field defects. It is notable, however, that IOP reductions from baseline to last visit among the 687 subjects evaluated by applanation tonometry were similar to those observed for the total population. Although the design did not include a washout period between termination of baseline therapy and initiation of latanoprost/timolol FC combination, this would not be expected to impact the long-term outcomes evaluated herein. The 24-month follow-up period may have been too short to detect changes in visual fields. Moreover, last visit data for individual progression measures reflected a time point prior to month 24 for between 15% (for cup/disc ratio data) and 32% (for rim area/volume/retinal nerve fiber layer data) of evaluable subjects. Strict adherence to study procedures and reporting requirements could not be affirmed given the large number of participating physicians and the prolonged follow-up period. Finally, while 17% of patients reported a change in iris color from baseline to month 24, evaluations relied on recollections of baseline color. The Ocular Hypertension Treatment Study [2] found that 17.1% of subjects prescribed a prostaglandin analogue for at least six months and 7.6% of those in the observation group reported a change in iris color, darkening of the eyelids, or growth of eyelashes.

## Conclusions

This 24-month study demonstrated that latanoprost/timolol FC effectively reduces IOP levels and is well tolerated in patients switched from other ocular hypotensive therapies for medical reasons. Investigator assessments showed optic disc parameters and visual field to be stable throughout the follow-up period.

## Competing interests

Dr. Guzy is an employee of Pfizer Pharma GmbH. Mr. Miller is an employee of Pfizer Limited, UK Specialty Care. The research was funded by Pfizer Pharma GmbH.

## Authors' contributions

OS participated in the study concept and design, analysis and interpretation of data, and critical revision of the manuscript for important intellectual content. BH participated in the study concept and design, acquisition of data, analysis and interpretation of data, and critical revision of the manuscript for important intellectual content. CZ participated in the analysis and interpretation of data, critical revision of the manuscript for important intellectual content, and study supervision. PM participated in the analysis and interpretation of data, and critical revision of the manuscript for important intellectual content. All authors read and approved the final manuscript.

## Pre-publication history

The pre-publication history for this paper can be accessed here:

http://www.biomedcentral.com/1471-2415/10/21/prepub
